# Serum Otolin-1 and Otoconin-90 are not elevated in vestibular migraine: a preliminary case-control study

**DOI:** 10.3389/fnins.2026.1768629

**Published:** 2026-02-27

**Authors:** Ange Li, Zhenyi Fan, Lulu Li, Xiaoxia Liu, Qiongfeng Guan, Weinv Fan, Yunqin Wu

**Affiliations:** 1 Department of Neurology, Ningbo No. 2 Hospital, Wenzhou Medical University, Ningbo, China; 2 School of Medicine, Shaoxing University, Shaoxing, China

**Keywords:** biomarker, differential diagnosis, otoconia, Otoconin-90, Otolin-1, vestibular migraine

## Abstract

**Objective:**

Vestibular migraine (VM) is a common cause of episodic vertigo but lacks objective diagnostic biomarkers. The symptomatic overlap of VM with benign paroxysmal positional vertigo (BPPV) and Meniere’s disease (MD) complicates differential diagnosis. Given that elevated serum Otolin-1 and Otoconin-90 (OC90) levels are established biomarkers in BPPV and MD, this study aimed to determine whether these otoconia-derived proteins are also altered in VM.

**Methods:**

In this case-control study, 40 patients with definite VM and 143 age- and sex-matched healthy controls were included. Serum samples were collected during the interictal period. Otolin-1 and OC90 levels were quantified using enzyme-linked immunosorbent assays.

**Results:**

No significant differences were found in serum levels of Otolin-1 and OC90 between groups. The median (IQR) Otolin-1 level was 215.5 pg/mL (127.5–314.9) in the VM group vs. 200.6 pg/mL (127.7–284.2) in controls (Cliff’s δ = 0.08, *p* = 0.526). Similarly, the median (IQR) OC90 level was 39.8 ng/mL (29.4–60.8) compared to 32.7 ng/mL (27.9–77.9) in controls (Cliff’s δ = −0.07, *p* = 0.659). No correlations were observed between protein levels and clinical features. However, within the VM group, serum Otolin-1 levels were highest within 1-week post-attack and declined thereafter, showing a significant negative correlation with time (*r* = −0.372, *p* = 0.018). A similar phase-dependent pattern was observed for OC90 levels across the VM subgroups (*p* = 0.017), though without a significant correlation with continuous time.

**Conclusion:**

These preliminary findings indicate that serum Otolin-1 and OC90 levels are not altered in patients with VM compared to healthy controls. Exploratory analysis revealed a phase-dependent decrease in Otolin-1 within the VM group post-attack. These results argue against significant structural damage to otoconia in VM and support a central/functional pathophysiology. While not positive diagnostic biomarkers, normal levels of these proteins may provide negative evidence to aid in differentiating VM from BPPV or MD, pending future validation.

## 1 Introduction

Vestibular migraine (VM) is a prevalent condition affecting approximately 1% of the general population, characterized by recurrent episodes of vertigo, often accompanied by migrainous features like photophobia, phonophobia, or headache. It is recognized as one of the most common causes of episodic vertigo encountered in both neurotology and headache clinics (Formeister et al., 2018). However, the diagnosis of VM remains clinically challenging, relying solely on established criteria in the absence of definitive objective biomarkers (Lempert et al., 2022). This challenge is compounded by significant symptomatic overlap with other vestibular disorders, particularly Meniere’s disease (MD) and benign paroxysmal positional vertigo (BPPV), leading to misdiagnosis rates exceeding 30% (Beh et al., 2019; Chen et al., 2022; Baron and Steenerson, 2025; Cantillo-Martínez et al., 2025). Thus, identifying objective tools to differentiate VM from these peripheral disorders is of high clinical priority.

The prevailing pathophysiological model of VM centers on central nervous system mechanisms, particularly dysregulation of vestibular and trigeminal pathways within the brainstem and cortex (Akerman et al., 2009). However, emerging evidence suggests potential peripheral vestibular involvement, specifically otolithic dysfunction. This is supported by the frequent observation of abnormal vestibular evoked myogenic potentials in VM patients and a higher incidence of comorbid BPPV (Huang and Young, 2025; Zaleski et al., 2015; Zaleski-King and Monfared, 2021; Mok et al., 2025). While clinical findings hint at a possible vulnerability of the otolithic organs in VM, blurring the traditional central-peripheral dichotomy, direct biochemical evidence of this damage remains elusive.

Otolin-1 and Otoconin-90 (OC90) are the primary organic matrix proteins essential for the nucleation, growth, and structural stability of the calcium carbonate crystals within the vestibular system. Both are synthesized by vestibular supporting cells and are crucial for maintaining otolithic mass and balance function (Bielak et al., 2023; Dong et al., 2025; Wang et al., 1998; Whitfield, 2025). These proteins can cross the blood-labyrinth barrier and are detectable in serum. Elevated serum Otolin-1 levels have been consistently reported in patients with BPPV during acute episodes (Wu et al., 2020; Aygun et al., 2024; Fan et al., 2022). Similarly, recent study indicates significantly higher levels of both Otolin-1 and OC90 in patients with MD (Han et al., 2024). Consequently, Otolin-1 and OC90 have emerged as promising biomarkers for peripheral otolithic damage. Whether VM, a condition with suspected peripheral involvement, is also characterized by alterations in these biomarkers remains a critical and unresolved question.

Given the clinical parallels, we hypothesized that VM might similarly involve subclinical otolithic pathology, reflected in elevated serum levels of Otolin-1 and/or OC90. To test this hypothesis, we conducted a preliminary case-control study to measure and compare serum levels of these proteins in well-defined patients with VM and healthy controls.

## 2 Materials and methods

### 2.1 Study design and participants

This case-control study was performed according to the principles of the Declaration of Helsinki and approved by the Ethics Committee of Ningbo No.2 Hospital (protocol KY-2017-014 and KY-2020-038). Written informed consent was obtained from all participants.

We enrolled consecutive patients diagnosed with definite VM at our Department of Neurology, between January 2017 and March 2023. Diagnosed was based on the International Classification of Headache Disorders, 3rd edition (ICHD-3) and the Bárány Society criteria, defined as: (1) at least five episodes with vestibular symptoms of moderate or severe intensity, lasting 5 min to 72 h; (2) current or previous history of migraine with or without aura; (3) one or more migraine features (headache, photophobia, phonophobia, visual aura) occurring during at least 50% of vestibular episodes; and (4) not better accounted for another vestibular or ICHD-3 diagnosis (Lempert et al., 2022).

Concurrently, sex-and-age matched healthy controls were recruited from the Comprehensive Health Screening Center.

Exclusion criteria for all participants included: (1) history of BPPV, MD, vestibular neuritis, labyrinthitis, head trauma, any headache disorder (for healthy controls) or any non-VM headache disorder (for VM patients), or otologic surgery; (2) Patients with VM, pure-tone audiometry was performed when clinically indicated (e.g., for tinnitus, aural fullness, or subjective hearing fluctuation); those with audiometrically confirmed sensorineural hearing loss were excluded; (3) major organ dysfunction (e.g., neoplasm, chronic renal failure, nephrotic syndrome, hepatic failure, severe cardiovascular or cerebrovascular diseases)

### 2.2 Clinical assessment and data collection

Demographic and clinical data collected from all participants included age, sex, body mass index, medication history, lifestyle factors, and comorbidities. For patients with VM, disease-specific characteristics were recorded, including headache and vertigo episode duration and frequency. Additionally, all patients with VM completed the following assessments: Visual Analogue Scale (VAS) for vertigo intensity, Headache Impact Test-6(HIT-6), Dizziness Handicap Inventory (DHI), Hamilton Anxiety Scale (HAMA), and Hamilton Depression Scale (HAMD).

### 2.3 Sample collection and laboratory analysis

Fasting venous blood samples were collected from all participants. For patients with VM, samples were drawn during the interictal period. Samples were centrifuged at 3,000 × g for 15 min at 4°C within 2 h. The serum was immediately aliquoted to avoid repeated freeze-thaw cycles and stored at −80°C until analysis.

Serum concentrations of Otolin-1 and OC90 were quantified using commercial enzyme-linked immunosorbent assay (ELISA) kits (Human Otolin-1 ELISA Kit, QY-E03713; Human OC90 ELISA Kit, QY-E03710; Qayee Bio, Shanghai, China) according to the manufacturer’s instructions. Briefly, target proteins were captured by plate-coated antibodies and detected using biotinylated detection antibodies, streptavidin-HRP, and TMB substrate. Absorbance was read at 450 nm on a Spectra Max Plus 384 microplate reader, and concentrations were interpolated from recombinant protein standard curves. All samples were analyzed in triplicate. The lower limit of detection and lower limit of quantification were 10 pg/mL and 25 pg/mL for Otolin-1, and 5 ng/mL and 10 ng/mL for OC90, respectively. The intra- and inter-assay coefficients of variation were below 10 and 15%, respectively.

To minimize batch effects, samples from VM patients and healthy controls were randomly distributed and analyzed within the same experimental run. All laboratory personnel were blinded to the clinical group assignments during the analysis.

### 2.4 Statistical analysis

Statistical analyses were conducted using SPSS Statistics version 22.0 (IBM Corp., Armonk, NY, United States) and R software (version 4.3.0). Normality of continuous variables was assessed using the Kolmogorov-Smirnov test. Continuous variables were presented as mean ± standard deviation (SD) or median with interquartile range (IQR), as appropriate. Categorical variables are presented as numbers and percentages. Comparisons between groups were performed using the student’s *t*-test (for normal continuous data), the Mann-Whitney U test (for non-normal continuous data) or the chi-square test (for categorical data). To explore potential phase-dependent effects, VM patients were categorized into three subgroups based on the time since the last vertigo attack (within 1 week, 14 weeks, and above 4 weeks). Inter-group comparisons of serum Otolin-1 and OC90 levels were performed using the Kruskal-Wallis H test. Correlations were assessed using Spearman’s rank correlation coefficient.

For the primary outcomes, we complemented the Mann-Whitney U test with nonparametric effect size measures to quantify the magnitude of between-group differences. The Hodges-Lehmann median difference with its 95% confidence interval (CI) was reported as a robust estimate of the location shift between groups. Cliff’s delta (δ) with its 95% CI was calculated as a probability-based measure of effect size, representing the likelihood that a randomly selected value from the VM group exceeds one from the control group. Confidence intervals for both statistics were generated using the bootstrap method (Bias-Corrected and Accelerated, BCa) with 5,000 resamples, which does not rely on distributional assumptions.

Given the exploratory nature of this study and the lack of prior data on effect sizes for these biomarkers in VM, the sample size was determined primarily based on feasibility and patient availability during the study period. A *post-hoc* power analysis was performed using G Power software (version 3.1.9.7). Given the observed small effect sizes (Cohen’s *d* = 0.193 for Otolin-1 and *d* = −0.159 for OC90), our sample sizes (VM: *n* = 40, Control: *n* = 143), and an alpha level of 0.05, the achieved statistical power was 28 for Otolin-1 and 22% for OC90. This indicates the study was underpowered to detect statistically significant differences of such small magnitude. A two-tailed *p* < 0.05 was considered statistically significant.

## 3 Results

### 3.1 Participants

Forty patients with definite VM and 143 healthy controls were included in this study. The study groups were demographically comparable, with no significant differences in age, sex distribution, or comorbidities (Table 1). The VM cohort had a median disease duration of 12.5 years and a median of 11 attacks per month. The interval from the current symptom onset to blood collection was categorized as: within 1 week (42.5%), 1–4 weeks (45.0%), and beyond 4 weeks (12.5%).

**TABLE 1:** Demographic and clinical characteristics of the participants.

Variable	Vestibular migraine (*n* = 40)	Healthy control (*n* = 143)	*p* value
Sex (female), *n* (%)	30(75.0%)	94(65.1%)	0.535
Age (years), mean ± SD	56.7 ± 13.4	59.3 ± 11.4	0.273
BMI (kg/m^2^), mean ± SD	23.2 ± 2.9	23.9 ± 3.2	0.191
Smoking, *n* (%)	9(22.5%)	36(25.2%)	0.728
Drinking, *n* (%)	7(17.5%)	26(18.2%)	0.921
Hypertension, n (%)	14(35.0%)	44(30.8%)	0.611
Diabetes, *n* (%)	6(15.0%)	21(14.7%)	0.961
Disease duration (years), median (IQR)	12.5(7–20)	–	
Vertigo frequency (episode/month), median (IQR)	11.0(7.0–16.7)	–	
Headache impact Test-6, median (IQR)	58.5 (51.2–63.0)	–	
Intensity of vertigo, median (IQR)	6(4–7)	–	
Dizziness handicap inventory, median (IQR)	46(24–65)	–	
Hamilton anxiety scale, median (IQR)	8(4–11.8)	–	
Hamilton depression scale, median (IQR)	6.5(4–11.7)	–	
Otolin-1 (pg/mL)	215.6 (127.5–314.9)	200.6 (127.7–284.2)	0.526
Otoconin-90 (ng/mL)	39.8 (29.4–60.8)	32.7 (27.9–77.9)	0.659

BMI, body mass index was defined as weight in kilograms divided by the square of height in meters. *p* < 0.05 was considered statistically significant.

### 3.2 Serum Otolin-1 and OC90 levels

**FIGURE 1:**
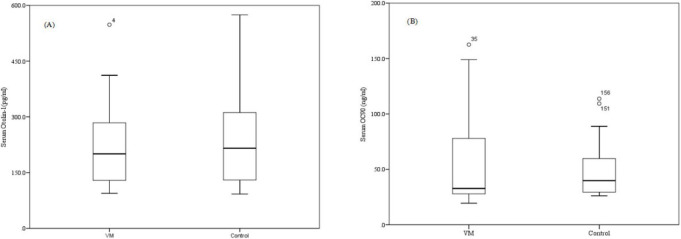
compares serum concentrations of Otolin-1 **(A)** and OC 90 **(B)** between the VM and healthy control, with data presented as median (IQR). No significant difference were observed in serum Otolin-1 levels between the VM group and the control group (*p* = 0.526), and similarly, serum OC 90 levels did not show a significant difference (*p* = 0.659). OC90, Otoconin-90; VM, vestibular migraine; IQR, interquartile range.

No statistically significant differences are observed between the VM and control groups for either protein (Figure 1). The median Otolin-1 concentration is 215.6 pg/mL (IQR: 127.5–314.9) in the VM group and 200.6 pg/mL (IQR: 127.7–284.2) in controls (Mann-Whitney *U* = 2,672, *p* = 0.526). The Hodges–Lehmann median difference is 14.9 pg/mL (95% CI: −27.88 to 57.73), with a Cliff’s delta of 0.066 (95% CI: −0.134 to −0.262), indicating a negligible effect size. Similarly, the median OC90 level is 39.8 ng/mL (IQR: 29.4–60.8) in the VM group and 32.7 ng/mL (IQR: 27.9–77.9) in controls (*U* = 2729.5, *p* = 0.659). The Hodges-Lehmann median difference is −1.26 ng/mL (95% CI: −20.67 to 17.88) with a Cliff’s delta of 0.046 (95% CI: −0.154 to 0.244).

When categorized by time since the last vertigo attack, serum Otolin-1 and OC90 levels differ significantly across the ≤ 1 week, 1–4 weeks, and > 4 weeks groups (*H* = 10.892, *p* = 0.004; *H* = 8.123, *p* = 0.017) (Table 2). Otolin-1 levels are highest within ≤ 1 week (median 271.63 pg/mL) and lowest after > 4 weeks (120.26 pg/mL; *p* = 0.028). OC90 shows a similar pattern, with significant differences for the ≤ 1 week vs. > 4 weeks (*p* = 0.049) and 1–4 weeks vs. > 4 weeks comparisons (*p* = 0.043). Furthermore, Otolin-1 levels correlate negatively with the continuous time variable (*r* = −0.372, *p* = 0.018), whereas no significant correlation is found for OC90 (*r* = −0.215, *p* = 0.186).

**TABLE 2:** Comparison of serum Otolin-1 and Otoconin-90 levels in patients with VM categorized by time since last vertigo attack.

Variable	Time of current symptom onset to blood collection	N (%)	Median (IQR)	Kruskal-wallis H	*p*-value	*Post-hoc* comparisons (*p*-value)
Otolin-1 (pg/mL)	≤1 week	17(42.5%)	271.6 (155.3–324.6)	10.892	0.004	≤1 week vs. 1–4 weeks: 0.196 ≤ 1week vs. ≥ 4 weeks: 0.028 1–4 weeks vs. ≥ 4 weeks: 0.112
	1–4 weeks	18(45.0%)	214.2 (140.9–259.2)			
	>4 weeks	5(12.5%)	120.3(97.1–149.5)			
Otoconin-90 (ng/mL)	≤1 week	17(42.5%)	47.8 (37.7–55.5)	8.123	0.017	≤ 1week vs. 1–4 weeks: 0.614 ≤ 1 week vs. ≥ 4 weeks: 0.049 1–4 weeks vs. ≥ 4 weeks: 0.043
	1–4 weeks	18(45.0%)	37.1(31.0–66.8)			
	>4 weeks	5(12.5%)	30.4 (29.1–30.8)			

Data are presented as median (interquartile range, IQR). Group comparisons were performed using the Kruskal-Wallis H test. *Post-hoc* pairwise comparisons were conducted using Dunn-Bonferroni method. *p* < 0.05 was considered statistically significant.

### 3.3 Correlation analysis

Spearman’s correlation analysis within the VM group reveals no significant correlations between serum levels of either protein and key clinical features of VM, including disease duration, vertigo frequency and intensity, or scores on the HIT-6, DHI, HAMA, and HAMD scales (all *p* > 0.05) (Supplementary Table 1).

## 4 Discussion

Our study provides a clear and clinically relevant negative finding: serum levels of Otolin-1 and OC90 do not differ significantly between patients with VM and healthy controls. Our findings challenge the hypothesis of subclinical damage to the otoconia themselves in VM, **as** reflected by the release of their constitutive matrix proteins, and reinforce the prevailing model of VM as a disorder primarily of central nervous system function (Akerman et al., 2009; Ceriani, 2024). It is important to note that this does not preclude functional alterations or damage to other components of the otolithic system.

A primary consideration is the limited sample size in the VM cohort and consequent low statistical power (22–28%). However, the observed the effect sizes were minimal (Cliff’s δ∼ 0.07), and the confidence intervals for the median differences were narrow and centered near zero. Therefore, while a larger sample would provide more precise estimates, it is unlikely to alter the core conclusion that substantial elevations akin to those seen in BPPV or MD are absent in VM.

The divergence of our findings from those observed in disorders with direct otolithic injury is both striking and informative. Significantly elevated serum Otolin-1 levels have been consistently reported in patients with idiopathic BPPV, particularly during acute episodes, and are associated with recurrence (Wu et al., 2020; Aygun et al., 2024; Fan et al., 2022). Similarly, in patients with MD, increases in both Otolin-1 and OC90 have been well documented (Han et al., 2024). Notably, although one prior study reported that serum otolin-1 levels were higher in patients with MD than in those with VM, this difference did not reach statistical significance in their cohort (Naples et al., 2022). Our study adds to this by directly comparing VM to healthy controls and including OC90, further supporting the lack of marked elevation in VM. The pattern observed in peripheral vestibulopathies is not universal. Patients with vestibular neuritis (VN), a condition primarily affecting the vestibular nerve, exhibit serum Otolin-1 and OC90 levels comparable to those of healthy controls (Wang et al., 2024). The consistent lack of elevation across both biomarkers in our VM cohort argues against a pathologic process analogous to BPPV or MD, aligning VM more closely with disorders lacking such structural pathology.

Interestingly, our exploratory subgroup analysis within the VM cohort reveals a time-dependent decline in serum Otolin-1 and OC90 levels following a vertigo attack. Although these levels remained within the normal range observed in healthy controls, this temporal pattern indicates a transient, state-dependent biological process. This finding aligns with the clinical observation that VM symptoms are episodic and may involve peripheral vestibular structures transiently, yet without leading to the persistent and marked elevation of otoconial proteins characteristic of BPPV or MD. Therefore, the dynamic pattern observed for Otolin-1 strengthens the concept that any peripheral involvement in VM is likely functional and reversible, rather than indicative of structural damage.

Our null finding solidifies the position of VM within the spectrum of migraine disorders, which are fundamentally diseases of brain function (Ceriani, 2024). The pathognomonic event in migraine is likely cortical spreading depression, which can dysregulate brainstem pathways that process vestibular and nociceptive signals (Akerman et al., 2009). This functional dysregulation provides a parsimonious explanation for why serum levels of Otolin-1 and OC90 remain unchanged in VM. In a patient with episodic vertigo, normal serum Otolin-1 and OC90 levels could therefore theoretically support a central diagnosis like VM over active peripheral otolithic disorders like BPPV or MD. However, the potential utility for differential diagnosis requires careful consideration. While a normal level may help differentiate VM from BPPV, the overlap in levels between VM and MD may limit its discriminatory power between these two entities. This proposed application remains speculative and hypothesis-generating, requiring validation through formal diagnostic accuracy studies.

While our data argue strongly against substantial structural damage to the otoconia, they do not preclude the possibility of more subtle, functional alterations in otolithic metabolism. A more plausible interpretation is that any otolithic involvement in VM is functional rather than structural, l, potentially arising from neurochemical imbalances or dysregulation of vestibular nerve activity (Zhai et al., 2025; Rahman and Luebke, 2024). Such functional disturbances, characteristic of the migrainous process, are unlikely to cause otoconia disintegration.

From a clinical perspective, our exploratory findings suggest a potential, albeit limited, diagnostic utility. For instance, in a patient presenting with episodic vertigo where VM and early MD are both considerations, a normal serum Otolin-1 level might incrementally favor a diagnosis of VM, whereas an elevated level would strongly suggest peripheral otolithic pathology. This illustrative scenario underscores that normal serum Otolin-1/OC90 levels could serve as a piece of supportive, negative evidence for a central disorder like VM. It is crucial to emphasize that this is not a standalone test and its value in distinguishing VM from MD may be lower due to potential overlap. An important future direction would be to evaluate these biomarkers in patients with episodic vertigo that does not meet current criteria for VM, MD, or BPPV, to explore their role in diagnosing atypical or overlapping presentations. Of course, this exploratory suggestion of differential utility would require validation through formal diagnostic accuracy studies with calculations of sensitivity, specificity, and predictive values.

### 4.1 Limitations and strengths

Our study has several limitations. First, the limited sample size in the VM cohort, which resulted in low statistical power (22−28%) as confirmed by a *post-hoc* analysis. While a larger sample is unlikely to alter the core conclusion regarding substantial elevation, this constraint necessitates that our findings be viewed as preliminary. Second, although our cross-sectional analysis identified a phase-dependent variation based on time since attack in Otolin-1 and OC90 levels, the single interictal time point of measurement limits our ability to delineate precise temporal dynamics. Future longitudinal studies with serial sampling are needed. Third, VM is a pathophysiological heterogeneous disorder. Future studies could investigate biomarker profiles across clinical subtypes and different disease phases (pre-ictal, ictal, post-ictal, interictal) to determine if biomarker profiles differ not only between patients but also within patients over time, potentially identifying distinct biochemical phenotypes.

## 5 Conclusion

In summary, this preliminary study found no significant alteration in serum Otolin-1 or OC90 levels in patients with VM compared to healthy controls. However, within the VM group, Otolin-1 levels varied with the time since the last attack, being higher in the acute phase. This suggests a transient, phase-dependent change that may reflect peripheral vestibular stress during attacks without evidence of persistent structural damage. These findings collectively argue against a pattern of sustained otolithic damage as seen in BPPV or MD, thereby reinforcing VM’s classification as a functional neurological disorder. While not a positive diagnostic test, normal levels of these proteins could potentially serve as supportive negative evidence in the differential diagnosis of episodic vertigo.

## Data Availability

The raw data supporting the conclusions of this article will be made available by the authors, without undue reservation.
