# Supplementary material for: Serum Otolin-1 and Otoconin-90 are not elevated in vestibular migraine: a preliminary case-control study
Source: Front Neurosci. 2026 Feb 27;20:1768629. doi: 10.3389/fnins.2026.1768629 (PMC12982408; doi:10.3389/fnins.2026.1768629)
Supplement: Supplementary file 1 [file Table_1.docx]

**Supplementary Table 1.** Spearman correlation coefficients between serum otoconia proteins and clinical variables in forty patients with vestibular migraine.

| Variable | Otolin‑1 | OC90 | Duration(years) | DHI | HAMA | HAMD | HIT‑6 | VAS | Frequency |
| --- | --- | --- | --- | --- | --- | --- | --- | --- | --- |
| Otolin‑1 | -- | 0.72** | –0.08 | –0.03 | –0.17 | –0.17 | 0.18 | 0.07 | –0.03 |
| OC90 | 0.72** | -- | –0.14 | –0.08 | –0.18 | –0.20 | –0.10 | –0.22 | –0.12 |
| Duration(years) | –0.08 | –0.14 | -- | 0.26 | 0.02 | 0.04 | 0.16 | 0.16 | 0.05 |
| DHI | –0.03 | –0.08 | 0.26 | --- | 0.74** | 0.63** | 0.27 | 0.04 | 0.12 |
| HAMA | –0.17 | –0.18 | 0.02 | 0.74** | -- | 0.84** | 0.18 | 0.18 | –0.06 |
| HAMD | –0.17 | –0.20 | 0.04 | 0.63** | 0.84** | -- | 0.19 | 0.11 | –0.09 |
| HIT‑6 | 0.18 | –0.10 | 0.16 | 0.27 | 0.18 | 0.19 | -- | 0.32* | 0.19 |
| VAS | 0.07 | –0.22 | 0.16 | 0.04 | 0.18 | 0.11 | 0.32* | -- | –0.15 |
| Frequency | –0.03 | –0.12 | 0.05 | 0.12 | –0.06 | –0.09 | 0.19 | –0.15 | -- |

DHI: Dizziness Handicap Inventory; HAMA: Hamilton Anxiety Scale; HAMD: Hamilton Depression Scale; HIT‑6: Headache Impact Test‑6; VAS: Visual Analogue Scale for vertigo intensity; Frequency: vertigo attack frequency (episode/month). * p < 0.05, ** p < 0.01 (two‑tailed).
